# *De novo* Assembly of the Grass Carp *Ctenopharyngodon idella* Transcriptome to Identify miRNA Targets Associated with Motile Aeromonad Septicemia

**DOI:** 10.1371/journal.pone.0112722

**Published:** 2014-11-19

**Authors:** Xiaoyan Xu, Yubang Shen, Jianjun Fu, Liqun Lu, Jiale Li

**Affiliations:** 1 Key Laboratory of Exploration and Utilization of Aquatic Genetic Resources, Shanghai Ocean University, Ministry of Education, Shanghai 201306, PR China; 2 E-Institute of Shanghai Universities, Shanghai Ocean University, 999 Huchenghuan Road, 201306 Shanghai, PR China; 3 National Pathogen Collection Center for Aquatic Animals, College of Fisheries and Life Science, Shanghai Ocean University, 999 Huchenghuan Road, 201306 Shanghai, PR China; Natural Resources Canada, Canada

## Abstract

**Background:**

*De novo* transcriptome sequencing is a robust method of predicting miRNA target genes, especially for organisms without reference genomes. Differentially expressed miRNAs had been identified previously in kidney samples collected from susceptible and resistant grass carp (*Ctenopharyngodon idella*) affected by *Aeromonas hydrophila*. Target identification for these differentially expressed miRNAs poses a major challenge in this non-model organism.

**Results:**

Two cDNA libraries constructed from mRNAs of susceptible and resistant *C. idella* were sequenced by Illumina Hiseq 2000 technology. A total of more than 100 million reads were generated and *de novo* assembled into 199,593 transcripts which were further extensively annotated by comparing their sequences to different protein databases. Biochemical pathways were predicted from these transcript sequences. A BLASTx analysis against a non-redundant protein database revealed that 61,373 unigenes coded for 28,311 annotated proteins. Two cDNA libraries from susceptible and resistant samples showed that 721 unigenes were expressed at significantly different levels; 475 were significantly up-regulated and 246 were significantly down-regulated in the SG samples compared to the RG samples. The computational prediction of miRNA targets from these differentially expressed genes identified 188 unigenes as the targets of 5 conserved and 4 putative novel miRNA families.

**Conclusion:**

This study demonstrates the feasibility of identifying miRNA targets by transcriptome analysis. The transcriptome assembly data represent a substantial increase in the genomic resources available for *C. idella* and will provide insights into the gene expression profile analysis and the miRNA function annotations in further studies.

## Background

Next-generation sequencing (NGS) -based RNA sequencing for transcriptome methods (RNA-seq) allow simultaneous acquisition of sequences for gene discovery as well as identification of transcripts involved in specific biological processes. This is especially suitable for non-model organisms whose genomic sequences are unknown [1], [2]. In addition, the dynamic range, sensitivity and specificity of RNA-seq also make it ideal for quantitatively analyzing various aspects of gene regulation [3]. These techniques do not require prior knowledge of genomic sequence and are much advanced in terms of time, cost, labor, amount of data produced, data coverage, sensitivity, and accuracy compared to traditional sequencing methods [4], [5].

The grass carp (*Ctenopharyngodon idella*) is one of the most important farmed fish species in China, with a cultural history dating back to the 7th century CE (Tang Dynasty) [6]. According to the FAO, the value of farmed *C. idella* reached more than 6.46 billion USD for a production of 5.03 billion tons in 2012, thus accounting for the highest production and third highest value of major cultured fish species worldwide at single species level [7]. Despite favorable growth traits, farmed *C. idella* are rather susceptible to various disease. Outbreaks of disease associated with bacteria such as *Aeromonas hydrophila* have caused high mortality, resulting in reduced production and considerable economic losses [8].

*A. hydrophila* is a causative agent of a wide spectrum of diseases in humans and animals [9]. While originally thought to be an opportunistic pathogen in immunocompromized humans, an increasing number of intestinal and extraintestinal disease cases suggest that it is an emerging human pathogen irrespective of the immune status of the host [10]. The pathogenesis, pathogenic mechanism, and virulence factors responsible for selected *A. hydrophila* infections in different species are not well understood [11]. *A. hydrophila* is a Gram-negative motile bacillus widely distributed in aquatic environments. It causes motile aeromonad septicemia (MAS), which results in great economic losses in worldwide freshwater fish farming [12]. Thus, more effective measures against *A. hydrophila* infection in fish are needed. Identification of differentially expressed genes (DGEs) following *A. hydrophila* infection is important for an improved understanding of fish MAS.

MicroRNAs (miRNAs) are 20–22 nt non-coding RNAs that play important roles in post-transcriptional gene regulation. In animal cells, miRNAs regulate their targets by translational inhibition and mRNA destabilization [13]. MicroRNAs (miRNAs) are key effectors in mediating host-pathogen interactions and constitute a family of small RNA species; they are considered a promising candidate for regulating the interaction between host and pathogen [14], [15]. Therefore, dissecting the biological functions of miRNAs may help us understand the pathogenic mechanism of motile aeromonad septicemia in *C. idella*. Many studies have identified miRNAs and mRNA transcriptome in fish species, like common carp [16], [17], nile tilapia [18], [19] rainbow trout [20], [21] channel catfish [22], [23] and silver carp [24], [25]. To thoroughly interpret the biological functions of these miRNAs, a first step is to predict their targets. Therefore, establishing a more powerful transcriptome data for target identification is preferred.

Although two parallel *C. idella* expressed sequence tag analyses have already been conducted using head kidney tissue [26], [27], the data presented here represent the first effort to analyze the transcriptome of *C. idella* affected by *A. hydrophila*. Two cDNA libraries from SG and RG *C. idella* used for our miRNA analysis were constructed and sequenced with Illumina Hiseq 2000. The obtained reads were assembled into transcripts and annotated by BLAST analysis against various databases before screening the results for differentially expressed genes and the prediction of miRNA targets. Our work will provide an approach to identify the target genes of miRNAs and to characterize their functional/regulatory network to increase our understanding of hemorrhagic septicemia outbreaks in *C. idella*.

## Materials and Methods

### Ethics statement

All handling of fishes was conducted in accordance with the guidelines on the care and use of animals for scientific purposes set up by the Institutional Animal Care and Use Committee (IACUC) of the Shanghai Ocean University, Shanghai, China. The IACUC has specially approved this study within the project “Breeding of Grass Carp” (approval number is SHOU-09-007).

### Sample collection

*C. idella* with an average weight of 50 g were cultured individually at the Wujiang National Farm of Chinese Four Family Carps (Jiangsu Province, China). Animals were raised at 28°C in 400-L aerated tanks for one week before the experiment and fed twice daily (in the morning and late in the afternoon) at a ratio of 5% of total biomass. Two groups (30 animals in each group) were maintained in two aquariums and intraperitoneally injected with *A. hydrophila* AH10 (Aquatic Pathogen Collection Center of Ministry of Agriculture, China) at a dose of 7.0×10^6^ cells suspended in 100 µl PBS per fish. All fish were observed every 4 h for any mortality and samples were taken until the termination of the experiment at 240 h post-challenge. *C. idella* died in the first 72 h post-challenge were classified as susceptible group (SG) for their high sensitivity to *A. hydrophila*, while the animals that survived over 240 h post-challenge were considered as resistant group (RG) [28]. Spleen and kidney samples were immediately snap-frozen in liquid nitrogen and stored at −80°C until further use.

### cDNA library construction and sequencing

Experimental protocols for the cDNA sequence were performed according to the manufacturer's technical instructions. The spleen and kidney tissues of randomly-selected three fish from both the susceptible and resistant groups were collected and, labeled as SG and RG, respectively. The RNA from the same tissue of three fishes of SG and RG *C. idella* was pooled with equal quantity for the construction of SG and RG cDNA libraries. The pooled total RNA was isolated from each spleen and kidney samples with TRIZOL reagent (Invitrogen, Grand Island, NY, USA). RNA integrity was confirmed using a 2100 Bioanalyzer (Agilent Technologies, Inc.) by RNA 6000 nano with a minimum RNA integrity number (RIN) value of 7.0. Poly (A) mRNA was purified from the total RNA using oligo (dT) magnetic beads. Equal amounts of the high-quality mRNA samples were obtained from each group for cDNA library preparation using the NEBNext Ultra RNA Library Prep Kit for Illumina (New England Biolabs, Ipswich, MA, USA) and purified using Agencount AMPure XP beads (Beckman Coulter, Krefeld, Germany) according to the manufacturer's recommendations. The concentration of the cDNA library was determined on an Agilent Technologies 2100 Bioanalyzer by Agilent DNA-1000. Libraries were sequenced, at the Novogene Bioinformatics Institute (Beijing, China) on an Illumina HiSeq 2000 instrument (Illumina, San Diego CA, USA) that generated paired-end reads of 101 nucleotides.

### Data processing, assembly, and functional annotation

Raw reads generated by Illumina Hiseq 2000 were then cleaned by removing the adaptor containing sequences, any ambiguous base>10% reads and low quality reads (1/2 reads with Q-value≤5) to get clean reads. Then, all clean reads were assembled using the *de novo* assembly program Trinity [29]: first, short reads were assembled into high-coverage contigs that could not be extended farther in either direction in a k-mer-based approach for fast and efficient transcript assembly. Then, the related contigs were clustered and a de Bruijn graph for each cluster was constructed. Finally, in the context of the corresponding de Bruijn graph and all plausible transcripts, alternatively spliced isoforms and transcripts were derived.

All assembled transcripts were compared with publicly available databases including Nr (NCBI non-redundant protein sequences), Nt (NCBI non-redundant nucleotide sequences) [30], KOG/COG (Clusters of Orthologous Groups of proteins) [31], Swiss-Prot (a manually annotated and reviewed protein sequence database) [32], KO (KEGG ortholog database) [33], Pfam (protein family) [34] and GO (Gene Ontology) (http://www.geneontology.org/). Nr, Nt, KOG/COG, Swiss-Prot, and KO used the BLASTx analysis with a cut-off *E*-value of 10^−5^, Pfam used Hmmerscan and GO used Blast2GO [35]. The best Blast hits from all Blast results were parsed for a homology-based functional annotation. For the nr annotations, the Blast2GO program was used to obtain GO annotations of unique assembled transcripts to describe biological processes, molecular functions, and cellular components.

### Differentially expressed genes between the SG/RG libraries

High-quality reads were mapped to reference sequences (unigenes from the transcriptome data of the cDNA library) using RSEM [36]. Gene expression levels were calculated using the fragments per kilo bases per million mapped reads (FPKM) method [37]. The calculation of unigene expression levels and the identification of unigenes that were differentially expressed between the libraries were performed by DEGseq [38] based on TMM normalized counts. The settings “q.value <0.005” and “|log2.Fold change.normalized|>2” were used as thresholds for judging significant differences in transcript expression. Differentially expressed genes across the samples were further annotated by GO and KEGG pathway analysis

### MiRNA target prediction

The SG and RG kidney miRNA-seq analysis were conducted in the same biological samples as mRNA-seq. Small RNA libraries were constructed using a Small RNA Cloning Kit (Takara). RNA was purified by polyacrylamide gel electrophoresis (PAGE) to enrich for the molecules in the range of 17–27 nt, then was ligated with 5' and 3′ adapters. The resulting samples were used as templates for cDNA synthesis followed by PCR amplification. The obtained sequencing libraries were subjected to Solexa sequencing-by-synthesis method. After the run, image analysis, sequencing quality evaluation and data production summarization were performed with Illumina/Solexa pipeline. The sequencing data was pretreated to discard low quality reads, no 3′-adaptor reads, 5′-adaptor contaminants and sequences shorter than 18 nucleotides. After trimming the 3′ adaptor sequence, sequence tags were mapped onto the transcriptome of *C. idella* using bowtie. Any small RNAs having exact matches to transcriptome of *C. idella* were used from further analysis. The mapped reads were compared to the miRBase (19.0) to annotate conserved miRNAs. To predict novel miRNAs, the miREvo [39] and mirdeep2 [40] were used.

Computational identification of differentially expressed miRNA targets was performed using the miRanda toolbox [41], using the complementary region between miRNAs and mRNAs and the thermodynamic stability of the miRNA-mRNA duplex. All mRNAs used for target prediction came from the differentially expressed unigenes obtained as described above. The miRanda toolbox employed a dynamic programming algorithm to search the complementary regions between the miRNA and the 3′-UTR of the mRNA, and the scores were based on sequence complementary as well as minimum free energy of RNA duplexes, and were calculated with the Vienna RNA package [42]. All detected targets with scores and energies less than the threshold parameters of S>90 (single-residue pair scores) and ΔG <−17 kcal/mol (minimum free energy) were selected as potential targets.

### Real time PCR validation

The sequencing results were validated by real time PCR using One Step PrimeScript miRNA cDNA Synthesis Kit for miRNA (TaKaRa) reversely transcribed, PrimeScript RT reagent Kit with gDNA Eraser (TaKaRa) for mRNA and SYBR *Premix Ex Taq* II (2x) (Takara) for qPCR according to the manufacturer protocols. Specific primer assays for *miR-21* [F: 5′-TAGCTTATCAGACTGGTGTTGGC-3′, R: Uni-miR qPCR Primer (TaRaKa)], *JNK1* (F: 5′- TGGTCAGAGGTAGTGTGTTG-3′, R: 5′-AGTTTGTTGTGGTCCGAGTC-3′) and *ccr7* (F: 5′-CAAGCCAAGAACTTTGAGAGG-3′, R: 5′-GGCATAAAGGCGAATGTTGTC-3′) were purchased from sangon biotech and real time PCR quantification was carried out in CFX96 Real-Time PCR System (Bio-Rad, CA, USA). To normalize the expression values, *miR-22a* for miRNA and *18s* for mRNA were used as housekeeping control [43], [44]. Expression levels were quantitatively analyzed using the 2^−△△CT^ method. One-way ANOVA tests were performed using SPSS 17.0 to determine significant differences. Each experiment was repeated in triplicates.

## Results and Discussion

Antagonistic bacteria, such as *A. hydrophila*, enhance non-specifically immune-related enzyme activities and disease resistance in *C. idella* and provide a theoretical basis for disease prevention in aquaculture. However, the molecular mechanisms of this disease are still far from fully understood. The identification and characterization of candidate genes involved in MAS would represent the first step in understanding the genetic basis of this process in *C. idella*.

### *De novo* assemblies and unigenes annotation

**Figure 1:**
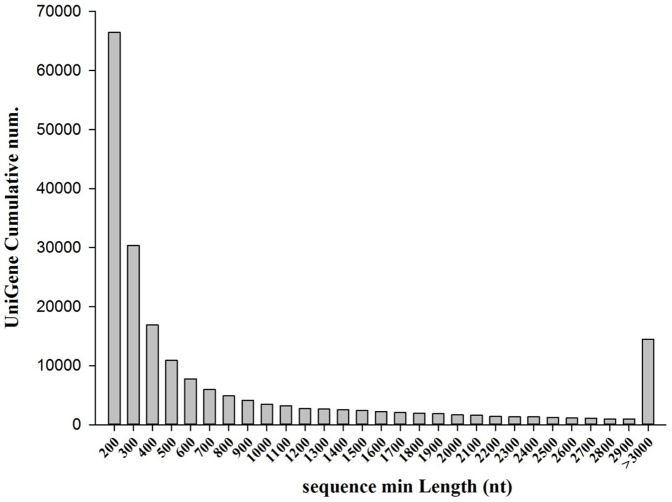
Length distribution of assembled unigenes in the sequenced cDNA library.

The sequencing generated 135.80 million raw reads. After trimming, 130.88 million clean reads remained, corresponding to 13.22 GB clean bases. The dataset of each sample, SG and RG, was represented by over 60 million clean reads, a read density sufficient for the subsequent quantitative analysis of genes. The raw sequencing reads have been submitted to the NCBI Short Read Archive under the accession number of SRR1124206 and SRR1125014. Then, all clean reads were assembled using a *de novo* assembly program Trinity [45]. These short reads were further assembled into 199,554 transcripts with an average length of 977 bp (Table 1). The size distribution of these transcripts ranged from 201 to 27,185 bp, of which 27,334 were larger than 2,000 bp (Figure 1). The assembled transcriptome data were deposited in NCBI's Transcriptome Shotgun Assembly (TSA) database under the accession numbers from SUB583458.

**Table 1:** Summaries of sequencing cDNA library.

Sample name	SG	RG
Total reads	73,063,654	62,737,669
Clean reads	70,210,307	60,668,815
Total mapped to unigenes readcounts	61,396,052.97	51,785,549.97
Reads length (bp)	101	
GC content (%)	48.43	47.13
Number of unigenes	199,554	
Total length of unigenes (bp)	195,075,872	
Mean length of unigenes (bp)	977	
N50 of unigenes (bp)	2,117	
Maximal length of unigenes (bp)	27,185	

### Annotation of predicted proteins

**Figure 2:**
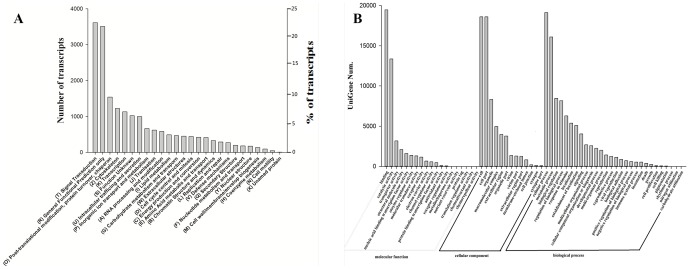
Functional annotations of the unigenes of *C. idella*. (A) KOG annotation. (B) Level 2 GO term distribution for the biological process, cellular component and molecular function categories.

A total of 61,373 distinct sequences (30.75% of the transcripts) matched known genes corresponding to 28,311 of the annotated proteins (Table 2, Table S1). An additional functional annotation of the unigenes of *C. idella* was performed searching for putative orthologs and paralogs within the KOG database [31]. A total of 16980 unigenes (27.7%) were assigned to 26 eukaryotic orthologous groups (Figure 2A). The category “signal transduction”, which contained 3,611 unigenes (21.27% of 16980 unigenes), was the largest, followed by the categories “general functional prediction only” (3506, 20.65%), “post-translational modification, protein turnover, chaperone” (1539, 9.06%) and “cytoskeleton” (1226, 7.22%).

**Table 2:** Statistics of the annotation results for the *C. idella* unigenes.

	All	Nr	Nt	Pfam	KOG	Swiss-Prot	KO	GO
Number of unigenes	61373	28311	46653	33013	16980	22674	27775	34207
% of unigenes	100	46.1	76.0	53.8	27.7	36.9	45.3	55.7

Nr: NCBI non-redundant protein sequences, Nt: NCBI non-redundant nucleotide sequences, Pfam: Protein family, KOG: Clusters of Orthologous Groups of proteins, Swiss-Prot: A manually annotated and reviewed protein sequence database, KO: KEGG Ortholog database and GO: Gene Ontology.

### GO annotation and KEGG pathway analyses

After GO annotation, *C. idella* transcripts could be assigned to three categories: biological processes, molecular functions and cellular components. Within the various biological processes, cellular processes (19,121 unigenes) metabolic processes (16,091) and biological regulation (8,463) were the most highly represented members (Figure 2B). Important functions, such as cell death (389) and immune system processes (540), were also identified in this category. Similarly, cell (18,586) as well as cell part (18,586) and binding (19,460) were the most represented sub-categories in the cellular component and molecular function categories, respectively.

Searching against the Kyoto Encyclopedia of Genes and Genomes Pathway database (KEGG) [33] revealed that 10,561 unigenes could be matched to 298 KEGG pathways. The most-represented pathways hierarchy 2 were the “infectious diseases” pathway (3210 unigenes) and the “signal transduction” pathway (2316) (Table 3). Some pathways related to immune system were also identified, such as the “Toll-like receptor signaling” pathway (110) [46] and the “chemokine signaling” pathway (224) [47] (Table 4).

**Table 3:** Top 10 list of the gene number of Pathway Hierarchy 2.

Pathway Hierarchy 2	Unigene Number
Infectious Diseases	3210
Signal Transduction	2316
Cancers	2235
Nervous System	1648
Immune System	1554
Neurodegenerative Diseases	932
Digestive System	875
Endocrine System	861
Cell Communication	859
Signaling Molecules and Interaction	763

**Table 4:** Top 10 list of pathways related to immune system.

Pathway Hierarchy 2	KEGG Pathway	Unigene Numbers
Immune System	Chemokine signaling pathway	224
Immune System	Leukocyte transendothelial migration	199
Immune System	T cell receptor signaling pathway	156
Immune System	Fc gamma R-mediated phagocytosis	155
Immune System	Natural killer cell mediated cytotoxicity	129
Immune System	B cell receptor signaling pathway	110
Immune System	Toll-like receptor signaling pathway	110
Immune System	Antigen processing and presentation	86
Immune System	Fc epsilon RI signaling pathway	84
Immune System	RIG-I-like receptor signaling pathway	67

### Digital gene expression library sequencing

Based on the transcriptome sequence data, two DGE libraries were constructed to identify the differentially expressed unigenes between the SG and RG samples. After removing low-quality reads, 70,210,307 and 60,668,815 clean reads were generated from the SG and RG libraries, respectively (Table 1). Among these clean reads, 61,396,052.97 of the SG and 51,785,549.97 of the RG readcounts were mapped to unigenes.

### Differential gene expression between the SG and RG libraries

**Figure 3:**
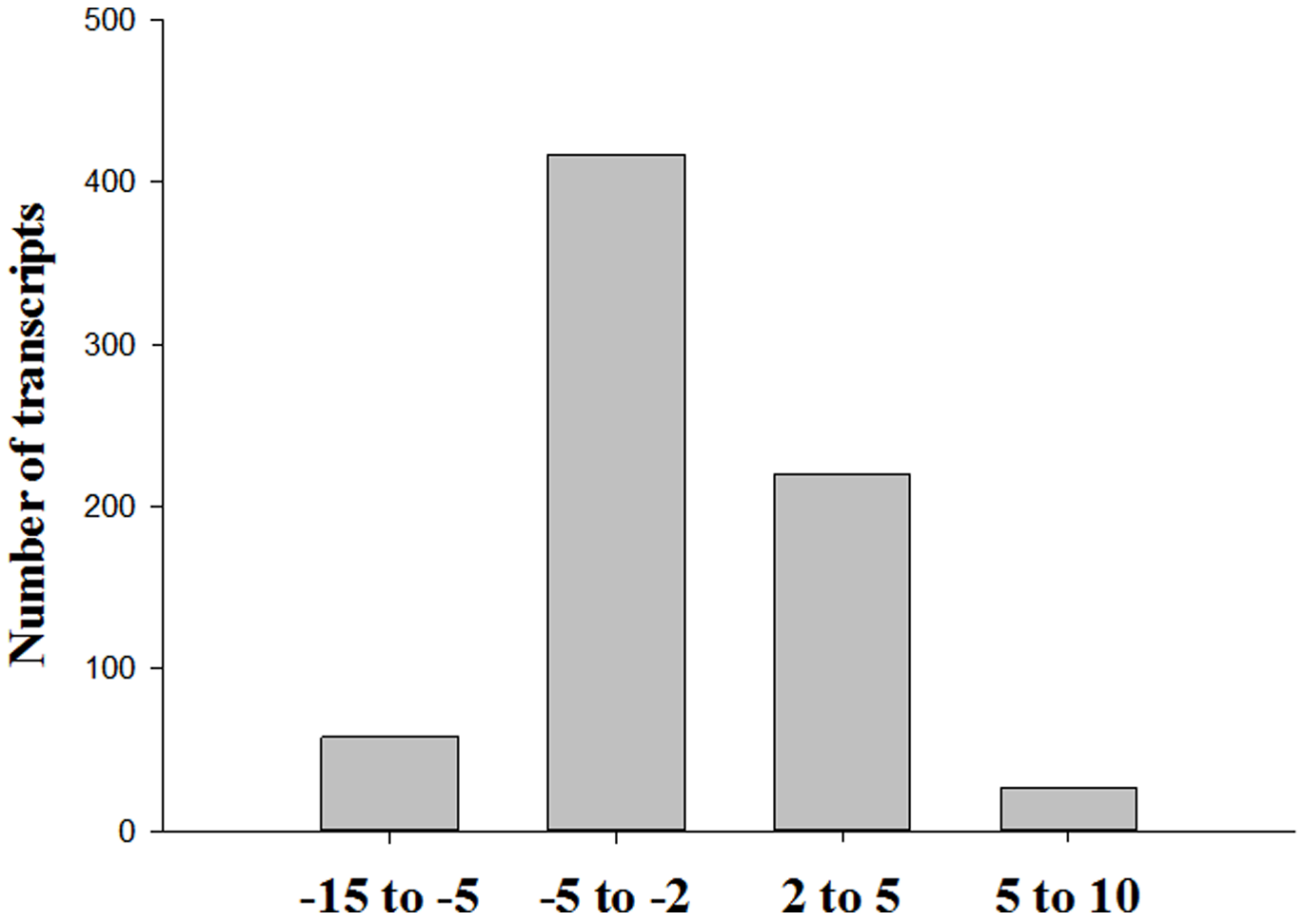
Number and Fold change distribution of differentially expressed genes between the SG/RG libraries.

The results suggest that the expression of 721 genes differed significantly between the SG and RG groups of *C. idella*. Of these genes, 475 were up-regulated and 246 were down-regulated in the SG samples compared to the RG samples (Figure 3 and Table S2). GO enrichment analysis of DEGs indicated that these genes were significantly enriched in oxidation-reduction processes (biological process), integral to membrane (cellular components), and protein binding (molecular function) (Table S3). Pathway enrichment analysis found the DEGs to be mainly enriched in complement and coagulation cascades, *Staphylococcus aureus* infection and porphyrin and chlorophy II metabolism (Table S4). Notably, several genes involved in the immune and inflammatory response were also identified, such as C-type lectin [48] and matrix metalloproteinase-9 [49].

### MiRNA target prediction

The identification of miRNAs and their targets is important for understanding the physiological functions of miRNAs and the functional roles of differentially expressed miRNAs between healthy and diseased fish. We were thus interested in predicting miRNA target genes involved in the immune response or immune system, according to the KEGG analysis. In a previous study, small RNA deep-sequencing data were aligned with miRBase 18.0 to search for known miRNAs with complete matches, namely, conserved miRNAs (data unpublished). Meanwhile, miRNAs predicted by miRDeep 2.0 that could form stable secondary structures, were identified as novel miRNAs.

We have used a single algorithm, miRanda [50] to predict miRNA targets. As miRNA binding to target 3′ UTR generally results in mRNA destabilization and degradation [51], we chose to narrow down potential targets to those showing differential expression in the opposite direction as the mRNA. This approach increases the strength to discover true target genes and functions affected by miRNA dysregulation. In total, 188 of the target genes predicted by miRanda were differentially expressed in the opposite direction in the target tissue. The identification of 188 unigenes (Table S5) as the predicted target genes of 5 conserved and 4 putative novel miRNA families (Table 5). The identified target genes involved in biological processes, molecular functions, and cellular components were defined using GO annotations. GO analysis demonstrated that these targets were involved in a broad range of physiological processes, including gene expression, transcription regulation, immune system processes, and responses to stress or stimuli (Table S6).

**Table 5:** Differentially expressed miRNAs in *C. idella* kidney between SG and RG.

miRNA	Sequence (5′-3′)	SG (TMP)	RG (TMP)	log2 (Fold_change) normalized	p-value
*let-7i*	UGAGGUAGUAGUUUGUGCUGUU	3419.67473	1475.401006	1.212751983	4.62E-174
*miR-142a-3p*	UGUAGUGUUUCCUACUUUAUGGA	4770.81118	9798.344345	−1.038303405	0
*miR-21*	UAGCUUAUCAGACUGGUGUUGGC	3431.54242	1640.502276	1.064719594	4.12E-142
*miR-217*	UACUGCAUCAGGAACUGAUUGG	240.9140969	2983.647091	−3.630486183	0
*miR-223*	UGUCAGUUUGUCAAAUACCCC	1460.912578	6800.683353	−2.218809871	0
*novel_115*	UGAAGGCCGAAGUGGAGA	3.560306851	17.95531055	−2.334337113	0.001253505
*novel_131*	UGCCCGCAUUCUCCACCA	7.713998177	40.28996513	−2.384869847	9.85E-07
*novel_154*	CCCAGCCAUAUUUGUUUGAAC	16.02138083	0	4.768565529	4.44E-05
*novel_3*	UGUUUCUGGCUCUGAUAUUUGCU	32.04276166	71.82124218	−1.164412111	8.07E-05

Searching against the KEGG indicated that 188 unigenes mapped to 48 KEGG pathways. 26 pathways were related to immune and diseases in all pathways (Table 6). These included the categories “Toll-like receptor signaling pathway”, “Fc epsilon RI signaling pathway”, “Chemokine signaling pathway”, “Fc gamma R-mediated phagocytosis”, “Antigen processing and presentation”, “Natural killer cell mediated cytotoxicity”, “T cell receptor signaling pathway” and “Complement and coagulation cascades” related immune functions. Toll-like receptor signaling pathway induce the expression of a variety of host defense genes. These include chemokine signaling pathway and other effectors necessary to arm the host cell against the invading pathogen [52]. Cytokine-cytokine receptor interaction play a pivotal role in the generation of immunological responses during bacterial infection [53]. This implicated functions that are likely regulated by miRNAs and suggests regulation of different pathways during immune activation in susceptible and resistant *C. idella.*

**Table 6:** 26 pathways were related to immune and diseases in all pathways.

KEGG Pathway	Pathway Hierarchy 2	Target gene of different expression miRNA
Toll-like receptor signaling pathway	Immune system	JNK, TLR5, TBK1
Hepatitis C	Infectious diseases: Viral	JNK, TBK1, LDLR
Salmonella infection	Infectious diseases: Bacterial	JNK, TLR5
Fc epsilon RI signaling pathway	Immune system	JNK, FYN
Tuberculosis	Infectious diseases: Bacterial	JNK, PLK3, CTSS
Influenza A	Infectious diseases: Viral	JNK, DNAJB1, TBK1
Measles	Infectious diseases: Viral	FYN, TBK1
MAPK signaling pathway	Signal transduction	JNK, MKP
Toxoplasmosis	Infectious diseases: Parasitic	JNK, LDLR
Chemokine signaling pathway	Immune system	CCR7, ADCY7, DOCK2
HTLV-I infection	Infectious diseases: Viral	JNK, ADCY7, SLC2A1
Herpes simplex infection	Infectious diseases: Viral	JNK, TBK1
Fc gamma R-mediated phagocytosis	Immune system	DOCK2
Prion diseases	Neurodegenerative diseases	FYN
Viral myocarditis	Cardiovascular diseases	FYN
Pathways in cancer	Cancers: Overview	JNK, SLC2A1
Transcriptional misregulation in cancer	Cancers: Overview	CCR7
Type II diabetes mellitus	Endocrine and metabolic diseases	JNK
Cytokine-cytokine receptor interaction	Signaling molecules and interaction	CCR7, TNFSF12
Antigen processing and presentation	Immune system	CTSS
Pertussis	Infectious diseases: Bacterial	JNK
Natural killer cell mediated cytotoxicity	Immune system	FYN
T cell receptor signaling pathway	Immune system	FYN
Chagas disease	Infectious diseases: Parasitic	JNK
Staphylococcus aureus infection	Infectious diseases: Bacterial	CFB
Complement and coagulation cascades	Immune system	CFB

**List of gene abbreviations**: JNK: c-Jun N-terminal kinase, TLR5: toll-like receptor 5, TBK1: TANK-binding kinase 1, LDLR: low-density lipoprotein receptor, FYN: tyrosine-protein kinase Fyn, CTSS: cathepsin S, PLK3: polo-like kinase 3, DNAJB1: DnaJ homolog subfamily B member 1, MKP: dual specificity MAP kinase phosphatase, DOCK2: dedicator of cytokinesis protein 2, ADCY7: adenylate cyclase 7, SLC2A1: MFS transporter, SP family, solute carrier family 2 (facilitated glucose transporter), member 1, CCR7: C-C chemokine receptor type 7, TNFSF12: tumor necrosis factor ligand superfamily member 12, CFB: component factor B.

**Figure 4:**
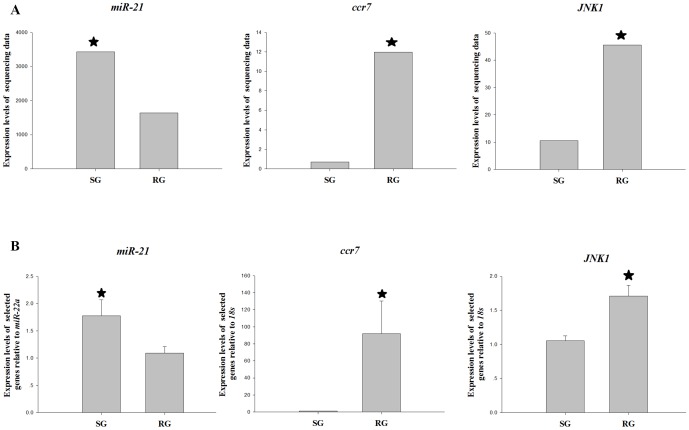
The expression analysis of selected genes from the expression profile by relative quantitative real-time PCR. A Transcriptome sequencing data, B Real-time PCR data. Increases and decreases in relative levels of transcripts with respect to the control 18s for mRNA and *miR-22a* for miRNA are shown. The settings “q.value <0.005” and “|log2.Fold change.normalized|>2” were used as thresholds for judging significant differences in transcript expression. One-way ANOVA tests were performed using SPSS 17.0 to determine significant differences for Real-time PCR data. Statistical significance of the relative expression ratio is indicated *.

Of particular interest to our study is the fact that several of the most highly expressed miRNAs in SG have been shown to have a role in immunity. let-7i and associated TLR4 expression are involved in cholangiocyte immune responses against *C. parvum* infection [54]. miR-21 targets multiple genes associated with the immunologically localized disease [55]. *miR-21* which is up-regulated in SG is predicted to target 28 differentially expressed genes (Table S7) in *C. idella*. Frequently represented in the top immune functions were protein kinase JNK1 (*JNK1*) and chemokine (C-C motif) receptor 7 (*ccr7*) (Table 6). *JNK1* and *ccr7* showed clearly down-regulated expression profiles in the SG samples compared to the RG samples (Figure 4A http://www.plosone.org/article/info%3Adoi%2F10.1371%2Fjournal.pone.0073506-pone.0073506.s003 ). The decreased expression profile of these targets in the susceptible samples supported our previous finding that the expression of *miR-21* was significantly up-regulated, with TMP of 3,432 and 1,642 in the SG and RG groups, respectively (Table 5). We validated the *miR-21*, *JNK1* and *ccr7* which had expression change in the sequencing data by performing real time PCR in the same samples used for the sequencing (Figure 4B).

JNK1 is involved in apoptosis, neurodegeneration, cell differentiation and proliferation, inflammatory conditions and cytokine production mediated by AP-1 (activation protein 1), such as Regulated upon activation normal T cell expressed and presumably secreted (*RANTES*), Interleukin 8 (*IL-8*), and Granulocyte-macrophage colony-stimulating factor (*GM-CSF*) [56]. It has been reported that JNK plays an important role in the innate immune response to microbial challenge [57], [58]. Most importantly, JNK1 serves as a negative regulatory factor for MAP kinase phosphatase 5 (*MKP5*) that plays an essential role in innate immune responses [59]. The chemokine receptor CCR7 acts as an important organizer of the primary immune response [60]. A previous study demonstrated a discrete CCR7 requirement in the activation of different T cell subsets during bacterial infection [61]. CCR7 is differentially regulated by macrophages in exposure to bacteria, as it is triggered by exposure to both Gram-negative and Gram-positive bacteria [62].

The discovery of microRNAs dramatically changed our perspective on eukaryotic gene expression regulation [63]. MicroRNAs play important gene-regulatory roles in animals and plants by pairing to the mRNAs of protein-coding genes to direct their posttranscriptional repression [64], [65]. The identification of miRNA target genes is an important step in understanding their role in gene regulatory networks. Most miRNA-associated computational methods comprise the prediction of miRNA genes and their targets, and an increasing number of computational algorithms and web-based resources are being developed to fulfill the needs of scientists performing miRNA research, like miRanda [42], TargetScan [66], RNAhybrid [67] and PicTar [68]. However, animal miRNA targets are difficult to predict since miRNA: mRNA duplexes often contain several mismatches, gaps, and G:U base pairs in many positions, thus limiting the maximum length of contiguous sequences of matched nucleotides [69]. The predicted interactions using these computational methods are inconsistent and the expected false positive rates are still high. Recently, several authors suggested integrating expression profiles from both miRNA and mRNA with *in silico* target predictions to reduce the number of false positives and increase the number of biologically relevant targets [70]. These methods have been shown to be effective in identifying the most prominent interactions from the databases of putative targets [71]. To minimize false positive rates in our study, the RNA-seq and miRNA-seq analysis were conducted in the same biological samples. Likewise, to reduce the number of putative target genes, the miRNA targets were predicted from differentially expressed genes. However, some false positive predictions proved inevitable. Further studies will focus on the experimental validation of the differentially expressed mRNA and miRNAs identified in this study. They are likely to be central regulators of the innate immune response to *A. hydrophila* and thus represent potential therapeutic targets or novel biomarkers of infection and inflammation.

## Conclusions

In this study, we used high-throughput sequencing data to characterize the transcriptome of *C. idella*, a species for which little genomic data are available. Further, DGE tags were mapped to the assembled transcriptome for further gene expression analysis. A large number of candidate genes involved in MAS were identified. This represents a fully characterized transcriptome, and provides a valuable resource for genetic and genomic studies in *C. idella*. Additionally, DGE profiling provides new leads for functionally studies of genes involved in MAS.

Finally, comparison with our previous miRNA profiling, this study strongly indicates that miRNA is a critical factor in determining mRNA abundance and regulation during MAS. Our on-going effort using experimental approach such as knock-down or over-express candidate miRNAs and mRNAs in vitro is expected to provide new evidence in understanding these regulatory mechanisms of MAS in *C. idella*.

## Supporting Information

Table S1 **Summary of unigene annotation against Nr, Nt, Pfam, KOG, Swiss-Prot, KO and GO database.**
(XLSX)

Table S2 **721 genes differed significantly between the SG and RG groups of** ***C. idella.***
(XLSX)

Table S3 **GO enrichment analysis of 721 genes differed significantly.**
(XLSX)

Table S4 **Pathway enrichment analysis of 721 genes differed significantly.**
(XLSX)

Table S5 **188 genes as the predicted target genes of 9 different expression miRNA between SG and RG groups of** ***C. idella.***
(XLSX)

Table S6 **GO enrichment analysis of 188 genes as the predicted target genes of different expression miRNA.**
(XLSX)

Table S7 **28 target gene information of** ***miR-21*** **.**
(XLSX)
